# The Terpolymer Produced by Azotobacter Chroococcum 7B: Effect of Surface Properties on Cell Attachment

**DOI:** 10.1371/journal.pone.0057200

**Published:** 2013-02-26

**Authors:** Anton Bonartsev, Sergey Yakovlev, Arasha Boskhomdzhiev, Irina Zharkova, Dmitrii Bagrov, Vera Myshkina, Tatiana Mahina, Elena Kharitonova, Olga Samsonova, Anton Zernov, Vsevolod Zhuikov, Yurii Efremov, Vera Voinova, Garina Bonartseva, Konstantin Shaitan

**Affiliations:** 1 Faculty of Biology, Moscow State University, Moscow, Russia; 2 A.N.Bach Institute of Biochemistry, Russian Academy of Sciences, Moscow, Russia; 3 Faculty of Physics, Moscow State University, Moscow, Russia; LAAS-CNRS, France

## Abstract

The copolymerization of poly(3-hydroxybutyrate) (PHB) is a promising trend in bioengineering to improve biomedical properties, e.g. biocompatibility, of this biodegradable polymer. We used strain Azotobacter chroococcum 7B, an effective producer of PHB, for biosynthesis of not only homopolymer and its main copolymer, poly(3-hydroxybutyrate-co-3-hydroxyvalerate) (PHB-HV), but also novel terpolymer, poly(3-hydroxybutyrate-co-3-hydroxyvalerate)-poly(ethylene glycol) (PHB-HV-PEG), using sucrose as the primary carbon source and valeric acid and poly(ethylene glycol) 300 (PEG 300) as additional carbon sources. The chemical structure of PHB-HV-PEG was confirmed by ^1^H nuclear-magnetic resonance analysis. The physico-chemical properties (molecular weight, crystallinity, hydrophilicity, surface energy) of produced biopolymer, the protein adsorption to the terpolymer, and cell growth on biopolymer films were studied. Despite of low EG-monomers content in bacterial-origin PHB-HV-PEG polymer, the terpolymer demonstrated significant improvement in biocompatibility in vitro in contrast to PHB and PHB-HV polymers, which may be coupled with increased protein adsorption, hydrophilicity and surface roughness of PEG-containing copolymer.

## Introduction

The last few decades have been characterized by intensive development of biomedical materials based on biodegradable polymers. Biodegradable polymers attract much attention in biology and medicine due to their broad application. Some medical applications of these polymers include medical implants for surgery, tissue engineering, novel drug dosage forms in pharmaceutics, novel materials for dentistry, etc. The widely known biodegradable polymers are polymers of lactic and glycolic acids (polylactides (PLA) and polyglycolides (PGA), respectively) and their copolymers, poly-ε-caprolactone, poly(orthoesters), poly-β-malic acid, poly(propylene fumarate), polyalkylcyanoacrylates, polyanhydrides, polyphosphazenes, poly(propylene fumarate), some natural polysaccharides (starch, chitosan, alginates, agarose, dextrane, chondroitin sulfate, hyaluronic acid), and proteins (collagen, silk fibroin, spidroin, fibrin, gelatin, albumin). Despite the large list of biodegradable polymers, many are produced by chemical synthesis (e.g., widely used copolymers of lactic and glycolic acids (PLGA)). However, chemical production of polymers for biomedical applications has a number of shortcomings, such as: the necessity for deep purification of chemical impurities, limited stereoregularity and synthesis of high-molecular weight polymers as well as limitations in accurate control of physicochemical properties of the produced polymers. On the other hand, the majority of natural biopolymers (e.g., chitosane, alginates, dextrane, collagen, etc.) are produced by isolation from plant or animal tissues that also have some key limitations, such as: the necessity of isolation and deep purification from plant or animal tissues, lack of control of the chemical structure and physicochemical properties of produced polymers, possible contamination by viral RNA/DNA, allergens and toxins, etc. [1], [2]. Therefore, some initially natural biopolymers, e.g. spidroin, were even produced by genetic engineering for medical application [3]. Hereupon, the improvement of polymer biomedical properties, e.g. biocompatibility, by blending or copolymerization is a promising trend in bioengineering.

Poly(3-hydroxyalkanoates) (PHAs) are biodegradable and biocompatible polyesters of bacterial origin. Unlike most biopolymers, PHAs are produced biotechnologically, which permits the control of chemical structure and physicochemical properties of the produced polymers during biosynthesis. PHB and its copolymers are natural biopolymers that display several unique properties, such as a high biocompatibility with mammalian cells, tissues and organs and the ability to biodegrade without forming toxic byproducts. [4], [5], [6]. PHB was the first member of the PHAs family to be identified. Now, over 300 PHA producers have been characterized; approximately 100 various hydroxyalkanoic acids in addition to PHB have been detected as components of PHAs [7], [8]. High levels of PHB accumulation in bacterial cells of biopolymer producers and the solubility of PHB in organic solvents make the process of isolation and deep purification of PHAs for biomedical applications relatively simple, while maintaining excellent quality. The widest field of PHB and its copolymers application includes surgical implants used in hernioplasty, dentistry, cardiovascular surgery and orthopedic surgery, etc. The biopolymers are used in development of biodegradable sutures, biodegradable screws and staples, periodontal membranes in dentistry, surgical meshes with biopolymer coatings, wound coatings, surgical patches for defects in the intestine, pericardium, or bone tissues and other tissues [5], [6]. Unfortunately, the PHB homopolymer has some physicochemical properties that limit its biomedical usefulness. Namely, the solution cast films of PHB have brittle properties, a high crystallinity degree, high hydrophobicity and low rate of biodegradation. These factors limit development, for example, in the formation of artificial blood vessels based on PHB biomaterial [9].

The improvement of PHB biocompatibility by copolymerization was developed by various chemical and biotechnological methods. However the chemical methods suffer from a series of shortcomings mentioned above and the most bacterial PHA producers can synthesize only PHB homopolymer or a certain PHB copolymer [1], [4], [5]. The effect of carbon nutrition conditions on PHA synthesis was actively studied in the context of the possibility to synthesize not only single-component, but also multicomponent PHAs to improve physicochemical and biomedical properties of biopolymers. It has been shown that the co-substrate, e.g. alkanoic acids, is the main factor in determining the PHA composition. As a rule, organic acids or alcohols with an odd number of carbon atoms are used as either primary or additional sources of carbon to produce copolymers by microbiological synthesis [7], [8]. For instance, when metabolized, valerate and propionate are transformed into a five-carbon compound, valeryl-CoA, which is used for synthesizing 3-hydroxyvalerate (HV) [10]. Introduction of 3-hydroxyvalerate (3HV) monomers into the chain of PHB leads to the production of copolymer PHB-HV with changed physico-mechanical properties (the melting temperature of PHB-HV is lower than PHB, it is more plastic, extensible, and resilient due to a decrease in the value of Young’s modulus) and higher biodegradability. Therefore, the PHB-HV polymer is well suited for broader applications. The impact strength, flexibility, and melting temperature of PHB-HV vary considerably when the 3HV mole fraction is present in the copolymer [11], [12], [13].

Moreover, Shi F. at al. suggested using not only monomeric organic acids or alcohols but also some polymers, e.g. polyethylene glycol (PEG), as additional carbon source for PHB copolymers biosynthesis [14], [15]. Polyethylene glycol (PEG), a neutral water-soluble polyether is relatively non-toxic to cellular systems and is absorbed into proteins and the phospholipid head group. PEG is used in processes such as protein modification, cell fusion and organ preservation, etc. [16], [17], [18]. In pharmacology and bioengineering, PEG is often used for chemical modification (PEGylation) of polymer nanoparticles, liposomes and biopharmaceuticals. PEGylation simply refers to the decoration of a polymer surface by covalently grafting, blending, or adsorbing PEG chains [19], [20]. The purpose of PEGylation of nanoparticles and liposomes is to create a barrier layer to block off opsonins present in the blood serum so that the particles can remain camouflaged or invisible to phagocytic cells and circulate longer [20]. PEGylation of some therapeutic proteins (e.g., interferon) significantly changes pharmacokinetic properties of these biopharmaceuticals [19]. PEGylation of biodegradable polymers (e.g., PLGA) is also used for improvement of polymer biocompatibility. It was shown that PHB/PEG blending improved cell compatibility and decreased blood coagulation and platelet adhesion to biopolymers compared to pure PHB. Improved cell- and hemo-compatibility was associated with increased hydrophilicity of PHB/PEG blends [21]. Chemical synthesis of PHAs-PEG copolymers has also been reported. PHB-PEG and poly(3-hydroxybutirate-co-4-hydroxybutyrate)-PEG copolymers were produced and their physicochemical properties were examined. However, the molecular weight (M_w_) of the copolymers was much lower than natural PHAs, which limits biomedical application of synthetic PHAs-PEG biopolymers [22], [23]. Unfortunately, the data concerning biocompatibility of PHAs-PEG copolymers or PHAs/PEG blends are often contradictory. Some investigators demonstrated that copolymerization or blending with PEG improves PHA biocompatibility [21], [23], while other researches claim that PEGylation causes impairment of PHA biocompatibility [24], [25], whereas the effect of biotechnological copolymerization of PHAs with PEG on polymer biocompatibility is even less clear.

Here, we produced PEG-containing PHB-HV copolymer by using Azotobacter chroococcum 7B and investigated how microbiological PEGylation of PHB-HV affect the physicochemical properties and biocompatibility of produced PHAs.

## Materials and Methods

### Materials

Poly(ethylene glycol) 300 g/mol (PEG 300), sodium salt of valeric acid (VA); components of growth media: K_2_HPO_4_•3H_2_O, MgSO_4_•7H_2_O, NaCl, Na_2_MoO_4_•2H_2_O, CaCO_3_, FeSO_4_•7H_2_O, sodium citrate, CaCl_2_, KH_2_PO_4_, sucrose, agar, phosphate-buffer saline (PBS); solvents for polymer isolation and purification: chloroform, isopropanol; liquids for surface energy measurment – Di(ethylen glycol), Poly(ethylene glycol) 400 g/mol and DMSO; All materials were purchased from Sigma-Aldrich and used as recommended by the manufacturer.

### Growth Conditions

A PHA producer *A. chroococcum* strain 7B, a non-symbiotic nitrogen-fixing bacterium able to overproduce PHB (to 80% of cell dry weight) was used [10], [26], [27], [28]. The strain was isolated from the wheat rhizosphere (sod-podzolic soil) and maintained on Ashby’s medium, containing 0.2 g/l K_2_HPO_4_•3H_2_O, 0.2 g/l MgSO_4_•7H_2_O, 0.2 g/l NaCl, 0.006 g/l Na_2_MoO_4_•2H_2_O, 5.0 g/l CaCO_3_, 20 g/l sucrose, and 20 g/l agar. All experiments were performed under laboratory conditions. For PHB synthesis in cells, the culture was grown in shaker flasks (containing 100 ml of the medium) at 30°C in Burk’s medium, containing: 0.4 g/l MgSO_4_•7H_2_O, 0.01 g/l FeSO_4_•7H_2_O, 0.006 g/l Na_2_MoO_4_•2H_2_O, 0.5 g/l sodium citrate, 0.1 g/l CaCl_2_, 1.05 g/l K_2_HPO_4_•3H_2_O, 0.2 g/l KH_2_PO_4_, and 17 g/l (50 mM) sucrose as the primary carbon source. For PHB copolymers biosynthesis, the additional carbon sources were added to the culture medium. As a 3HV precursor in the PHB-HV copolymer chain, VA was added as sodium salts at a concentration of 20 mM after 12 h incubation of the culture. The concentration of 20 mM VA and adding time were selected as optimal for production polymer with maximal 3HV content in produced copolymer [10]. PEG 300 was added at 18 h in the medium at a concentration of 150 mM. It was shown that further increases in PEG concentration inhibited growth, reduced PHA production, molecular weight and 3HV incorporation. Thus, we used the maximal optimal concentration of VA and PEG [15]. The experiment was performed for 72 h. Optical density was controlled by nephelometry. To control strain growth and polymer accumulation in cells a Biomed 1 (Biomed, Russia) light microscope was used. The parameters of the copolymers biosynthesis including biomass yield and polymer yield were determined according to [10], [26].

### Production of Highly Purified Biopolymers from Bacterial Biomass

The polymer isolation from *A. chroococcum* for biocompatibility study comprised the following stages: (1) polymer extraction with chloroform in a shaker for 12 h at 37°C; (2) separation of polymer solutions from cell debris by filtration; (3) polymer precipitation from chloroform solution with isopropanol. The purification procedure comprised the following repeated stages: 1) dissolution of isolated polymer in chloroform up to concentration of approximately 0.5 g/l, 2) supplying polymer solution in chloroform to isopropanol evenly for polymer separation, and 3) filtration of precipitated polymer flakes. The cycles of polymer purification were repeated for 4–5 times to remove any additives and contaminants. This method allows producing polymer with >99,5% purity for biomedical application as described in our invention [29]. The produced and purified polymer was dried at 60°C to obtain polymer beads [10], [26], [29].

### Molecular Weight Determination

Molecular weights of PHB, PHB-HV and PHB-HV-PEG were determined by gel permeation chromatography (GPC) using a Waters 1525 pump, connected to four Waters styragel columns (Styragel HT 6E, 4.6×300 mm) placed in series. The detection system consisted of a Waters 2414 differential refractive index detector and a UV detector. Chloroform was the eluent, at a flow rate of 1.0 mL/min. Typical sample volumes were 50 µL at a polymer concentration of 2 mg/mL. Narrow polydispersity polystyrene standards (Sigma-Aldrich, USA) were used to generate a universal calibration curve, from which the molecular weights were determined, after correcting for flowrate variations based on the elution volume of the flow-rate marker [30]. The M_w_ determined by GPC was correlated with data estimated by viscosimetry: the viscosity of the polymer solution in chloroform was measured at 30°C on an RT RheoTec viscometer (RheoTec, Germany); the molecular mass was calculated using the Mark-Kuhn-Houwink equation according to [26].

### Study of the Polymer Composition by Nuclear Magnetic Resonance (NMR)

**Figure 1:**
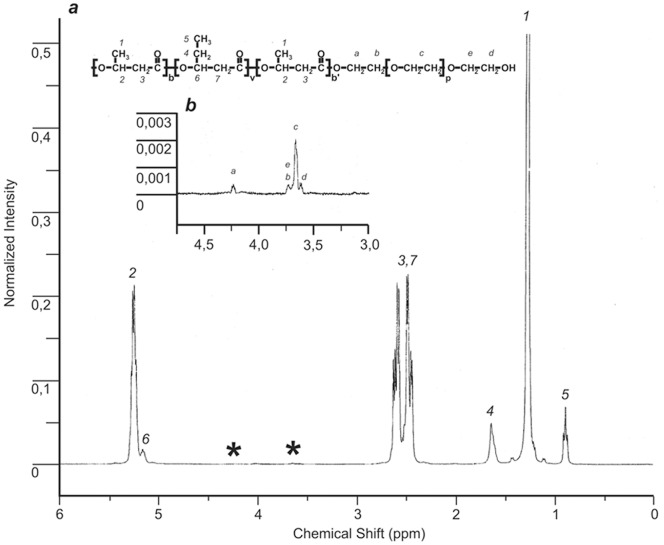
^1^H-NMR spectra of the PHB-HV-PEG copolymer. ^1^H-NMR spectra of the PHB-HV-PEG copolymer: (a) PHB chain: 1 is CH_3_(s), 2 is CH(b), 3 is CH_2_(b), PHV chain: 4 is CH_2_(s), 5 is CH_3_(s), 6 is CH(b), 7 is CH_2_(b), s is a side chain, and b is a polymer backbone; * see zoomed graph section on (b); (b) PEG chain: a is linking -O–CH_2_ (4.24 ppm), b is following CH_2_ (3.73), c is integral signal from backbone [-O–CH_2_–CH_2_-] group (3.66 ppm), e and d are tail –CH_2_- (3.70 ppm) and –CH_2_-OH (3.61 ppm) groups, respectively.

Proton (^1^H) NMR spectra of PHB and its copolymers solutions in deuterated chloroform were recorded in an MSL-300 (Bruker, Germany) spectrometer at a working frequency of 400 MHz. Chemical shifts in parts per million (ppm) were measured from 0.00 ppm relative to the signal of chloroform-d (CDCl_3_) residual protons, 7.27 ppm. The experimental parameters were as follows: 1% (w/v) polymer in chloroform-d, 313 K, 2.5 s acquisition time, and 4000 Hz spectral width. The percent content of elementary 3HV elements in the PHB–HV copolymer was calculated according to the ratio of the integral signal intensity from the 3HV methyl group (0.89 ppm) to the sum of integral signal intensities from the methyl groups of 3HV and 3-hydroxybutyrate (1.27 ppm) (Figure 1). The percent content of elementary EG elements in the PHB-HV-PEG copolymer was calculated according to the ratio of the sum of integral signal intensities from EG–CH_2_– groups (3.61, 3.70, 3.66, 3.73, 4.24 ppm) to the sum of integral signal intensities from the methyl group of 3-hydroxybutyrate (1.27 ppm) (Figure 1) [10], [26].

### Polymer Films Preparation

PHB, PHB-HV and PHB-HV-PEG films were prepared by casting a 3 wt. % chloroform solution of the polymers onto a glass Petri dish. After slow evaporation of chloroform, the remaining solvent in the films was removed by drying the films under vacuum at 50°C for two days. The thickness of polymer films was 50±5 µm [31].

### Crystallinity Degree of Biopolymers Measurement by Differential Scanning Calorimetry (DSC)

The PHB, PHB-HV and PHB-HV-PEG thermal properties were measured by means of differential scanning calorimetry using a DSC 204 F1 Phoenix (Netzsch, Germany) equipment. About 1–4 mg of polymer film was sealed in a 25 *µ*L aluminium crucible. The samples were heated from 25 to 200°C at a heating rate of 10°C/min in nitrogen atmosphere. Netzsch calibration set (KNO_3_, In, Bi, Sn, Zn, CsCl, Hg, C_6_H_12_ high purity samples) was used for precise temperature and enthalpy calibrations in temperature range −100°C –600°C according to the manufacturer instructions [32]. The onset and peak temperature of the change in heat capacity was designated as the T_m_^onset^ and T_m_^peak^ melting points. The crystallinity of PHB component (*X_c_*) can be calculated by the following [33]:
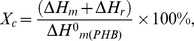
where Δ*H_r_*, Δ*H_m_*_._ are the enthalpy contributions caused by recrystallization and melting of investigated sample, respectively, Δ*H^0^_m(PHB)_* is the theoretical value for the thermodynamic melting enthalpy, which would be obtained for a 100%-crystalline PHB sample (146.6 J/g) [34]. All calculations were performed for the first heating cycle [30], [33].

### Contact Angle Tests

The hydrophilicity of polymer surface was evaluated by measuring the water contact angle formed between water drops and the “smooth” surface of the samples using a Contact Angle Meter 110 VAC (Cole-Parmer, USA). For this purpose, a drop of 10 µl of milliQ water was mounted on the surface with a microsyringe and quickly measured by the Contact Angle Meter. The advancing contact angle was measured for 8 drops on both sides at a temperature of 25°C, and the average was calculated from the data. The apparent contact angle can be measured exactly (accuracy of the method is 0.1%), but as a result of the roughness of the sample a typical statistical error was in the range of 1–2%. For the surface energy calculation contact angles of three more liquids (di(ethylen glycol), poly(ethylene glycol) 400 g/mol and DMSO) were measured as described before. The surface energy of test films was determined by geometric mean model [35], [36].

### Water Absorption

Films were cut into 10 × 10 mm samples and immersed in deionized water at 37°C. At predetermined time intervals, hydrated samples were picked up and weighed after the surface water was blotted away with Kimwipes. The water contents were then calculated on the basis of the weight difference of the film before and after swelling. The percentage of water uptake was calculated using the following equation
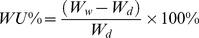
where WU% is water uptake (%), W_d_ and W_w_ are the weights of the sample film before and after being immersed in water, respectively [22].

### Atomic Force Microscopy

Microphotographs of the surface of PHA films were obtained be means of atomic force microscopy (AFM). The AFM imaging was performed with Solver PRO-M (Zelenograd, Russia). For AFM imaging a piece of the PHB film (∼2 × 2 mm^2^) was fixed onto a sample holder by double-sided adhesive tape. Silicon cantilevers NSG11 (NT-MDT, Russia) with a typical spring constant of 5.1 N/m were used. The images were recorded in semi-contact mode, a scanning frequency of 1–3 Hz, scanning areas from 3 × 3 to 20 × 20 µm^2^, and topography and phase signals were captured during each scan. The images were captured with 512 × 512 pixels. Image processing was carried out using Image Analysis (NT-MDT, Russia) and FemtoScan Online (Advanced technologies center) software.

Two quantitative parameters of roughness have been calculated to describe film surfaces. These include the average roughness, R_a_

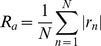
and the root mean square roughness, R_q_.



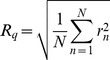



These parameters were calculated by three scan areas of 20 × 20 µm^2^ (512 × 512 points). Additionally, several scans at higher resolutions (e.g., 5 × 5 µm^2^ (512 × 512 points)) were obtained for each sample for more detailed description of the polymer surface [31].

### Protein Adsorption

The polymer films were incubated in Dulbecco’s Modified Eagle Medium (DMEM) containing 10% (v/v) fetal bovine serum (Invitrogen, USA), at 37°C for 24 h. After incubation, the samples were incubated in a buffer constituted by TRIS 10 mM, EDTA 1 mM and SDS 0.1% (v/w); the samples were mixed for 6 h at 3–4°C. This procedure permits to remove all proteins. In this way, the proteins adsorbed on the surface were removed from the sample and were determined by protein assay using Bradford Reagent (Sigma-Aldrich, USA) with a spectrophotometer Ultrospec 1100 pro (Amersham Biosciences Corp., USA). The experimental data are presented as the amount of protein adsorbed per unit surface area (cm^2^) of polymeric membranes [33], [37]. To visualize adsorbed protein on polymer film surface FITC-BSA (Sigma-Aldrich, USA) was used. Protein adsorptions by intensively washed (using MilliQ water) and dried polymer films were investigated by incubating the films in a solution of FITC-BSA in 10 mM TRIS for 2 h at 37°C. The analyses of adsorbed protein on the “smooth” surface of polymer films were carried out by fluorescence microscopy using Axiovert 200M fluorescent microscope with a digital AxioCam camera running the Zeiss LSM Image Browser 4.2.0 software (Carl Zeiss MicroImaging GmbH, Germany).

### Fibroblast Culture

The 3T3 murine fibroblast cells were used for polymer biocompatibility testing [3], [38], 39. The cells were cultivated in DMEM (Dubecco’s Modified Eagle Medium, Invitrogen, USA) with high glucose content (4.5 g/l) supplemented with 10% fetal calf serum (FCS), 100 IU/ml penicillin, and 100 *µ*g/ml streptomycin solutions (Invitrogen, USA). Cells were incubated at 37°C in a humidified 5% CO_2_ atmosphere and the medium was changed every day. Fibroblasts were released before confluence with trypsin-versen solution (0.05% (v/w) trypsin and 0.02% (v/w) EDTA in PBS) (Serva, Germany) and counted with Coulter Counter Z1 (Beckman Coulter, USA) [37].

### Cell Proliferation Studies

To analyze polymer biocompatibility cell attachment and proliferation on PHB, PHB-HV and PHB-HV-PEG polymer films were studied. The initial cell attachment to material surface and subsequent cell proliferation on (in) the material show integral indirect data on material properties that can be used for evaluation of living cells compatibility with the examined material: the biochemical reactivity of the material, the release of toxic products from the material, the availability of surface morphology of the material for cell growth, the biophysical surface properties (e.g. charge, hydrophilicity) of the material etc. Therefore, the cell viability tests for in vitro cell attachment and proliferation on the various materials are widely used to analyze biocompatibility of these materials [1], [2], [3], [6], [21], [24], [25], [33], [37], [40]. Eight samples for each polymer were placed in 96-well tissue culture plates and a cell suspension of 5000 cells/ml was directly seeded on every sample. Polymer films were placed in the wells with the “rough” surface upwards. The same amount of cells was plated in six empty polystyrene wells for each plate as a negative control. Plates was incubated for 24, 48, 72 and 96 h. Cell proliferation and viability were measured by the cell proliferation reagent based on the cleavage of the tetrazolium salt to soluble formazan salt by mitochondrial activity of viable cells (XTT Cell Proliferation Kit, Biological Industries, Israel). At the end of the experimental time, polymer films with attached cells were gently and quickly transferred from wells of incubated tissue culture plate to respective wells of new plate with preliminarily added 100 *µ*l fresh medium. Then 50 *µ*l XTT reagent solution was added to the cell monolayers on polymer films in each well, and the multi-well plates were incubated at 37°C for a further 4 h. Polymer films were removed and samples were quantified spectrophotometrically at 450 nm with reference wavelength at 640 nm. Viable cell numbers on films were then determined from the standard curve based on their XTT absorbency. Results were reported as optical density (OD) [37], [40].

### Statistical Analysis

Statistical evaluation of data was performed using the software package SPSS/PC+ Statistics™ 12.1 (SPSS). After verifying that the data were normally distributed and showed a homogeneity of variance, the non-parametric Kruskal–Wallis test was used to highlight any significant difference for *in vitro* and *in vivo* results between tested polymers for each experimental time by applying the following comparisons: PHB, PHB-HV and PHB-HV-PEG *versus* PLA; PHB-HV and PHB-HV-PEG *versus* PHB; PHB-HV-PEG *versus* PHB-HV. The Mann–Whitney *U*-test was used to compare results between experimental times for each tested polymer. Data were reported as the median ± SD at a significance level of *P<*0.05.

## Results and Discussion

### PHB-HV-PEG Copolymer Biosynthesis

Data on PHB and its copolymers biosynthesis by the *A. chroococcum* 7B culture grown in a medium containing sucrose as the primary carbon source and supplemented with valeric acid and PEG 300 as additional carbon sources are listed in Table 1. The data indicates that the combined addition of both VA and PEG caused considerable inhibition of cell growth, decrease of polymer content in cells, and consequently, a decrease in polymer yield in comparison with sole VA addition. Earlier, we have shown that adding various carboxylic acids (propanoic, hexanoic, heptanoic etc.,) to the culture medium also resulted in the inhibition of strain growth and polymer accumulation [10]. However, the effect of combined addition of VA and PEG on polymer molecular weight was much greater. Combined PEG 300 and VA addition to the medium resulted in a 11-fold drop in the molecular weight of produced polymer in comparison with 22% decrease in case of VA sole addition (Table 1). Earlier, in a serious of studies, PEG was also effectively used to decrease molecular weight of PHAs produced by *Ralstonia eutropha* [14], [15], *Alcaligenes latus* [41], [42], and *Pseudomonus oleovorans* [43].

**Table 1:** Physico-chemical properties of biopolymers: PHB, PHB-HV, and PHB-HV-PEG.

Polymers	Growth conditions and characteristics			Composition in polymer			Physico-chemical properties			
	Substrates (concent-ration, mM)	Bio-mass yields (g/l±SD)	Total PHA content, (wt.% ±SD)	HV content,mol%	PEG content,mol%	Molar ratioPEG/PHB	Mole-cularWeight(M_w_, ×10^3^)	M_w_/M_n_	Meltingtempera-ture,onset andpeak(T_m_^onset^/T_m_^peak^,°C)	Crystal-linity(*X*_c_, %)
PHB	Sucrose (50 mM)	4.7±0.4	81.2±4.2	0	0	0	1630	1.34	134.3/180.9	67.7
PHB-HV	Sucrose (50 mM)+VA (20 mM)	3.5±0.3	70.5±3.5	21.3	0	0	1270	1.46	128.2/174.7	50.0
PHB-HV-PEG	Sucrose (50 mM)+VA (20 mM)+PEG 300 (150 mM)	3.2±0.5	66.7±3.1	7.5	0,23	0,67	147	2.21	131.2/178.1	61.0

When sucrose is used as the sole carbon source for biopolymer synthesis, the PHA formed by *Azotobacter chroococcum* was a high molecular weight PHB (up to 1600 kDa) [10], [27]. Earlier, we have shown that 3HV is incorporated into the PHB–HV copolymer when using valeric and propanoic acids as additional carbon sources. The maximal 3HV content (21.6 mol %) in the copolymer was obtained when using 20 mM VA [10]. It was shown for bacteria of the genus *Azotobacter* that valeric acid is incorporated into the copolymer via the β-oxidation pathway: VA → valeryl-CoA → 3-ketovaleryl-CoA → D-3-hydroxyvaleryl-CoA → 3HV. In this case, sucrose was used as the main carbon source [44]. Early we confirmed 3HV incorporation into copolymer chains via ^1^H-NMR. The ^1^H-NMR spectrum of PHB-HV displays the signal of the 3HV methyl group at a chemical shift of 0.89 ppm versus the spectrum of the PHB homopolymer, which lacked this signal. The analysis of ^1^H-NMR spectra indicated that the copolymer is a multi-block copolymer, because the signal power of a proton of an esterified β-carbon group is directly proportional to signals of the 3HV methyl group at 0.89 ppm and the 3-hydroxybutyrate methyl group at 1.27 ppm [10].

Our data indicates that the combined use of additional carbon sources, VA and PEG 300, leaded to PHB-HV-PEG copolymer production by *Azotobacter chroococcum 7B*. Addition of PEG-300 (150 mM) to the growth medium simultaneously with VA also generated PEG incorporation into the PHA polymer, which was confirmed by ^1^H-NMR spectroscopy of PHB–HV-PEG copolymer, as shown in Figure 1a, b. Five weak ^1^H NMR signals at 3.66 ppm (the highest signal) and at 3.61, 3.70, 3.73, and 4.24 were observed that correspond to protons of EG repeat units. The signals at 4.24 and 3.73 ppm were assigned to protons a and b, respectively, of esterified PEG chain segments; peaks at 3.61 and 3.70 ppm were due to protons e and d of terminal free hydroxyl EG units. As seen in Figure 1b, the highest peak was the sum of signals from protons of median EG units of PEG. PHB and PHB-HV formed in the absence of PEG did not show ^1^H NMR signals in the 3.6–3.8 ppm spectral region [10]. The above results are consistent with the formation of PHA chains that are covalently linked at the carboxylate chain terminus to PEG chain segments. Thus, obtained copolymer is di-block copolymer of multi-block copolymer PHB-HV and PEG, where PEG is attached only to one end of the PHB-HV chain. The value of EG monomers incorporation in PHA (0.23 mol%) indicates that there are 0.67 molecules of PEG-300 per 1 molecule of PHB-HV (M_w_ = 1.47×10^5^) or two of three PHB-HV molecules were PEGylated. We have introduced this dimensionless parameter, the PEG/PHB-HV molar ratio, as the number of PEG molecules divided by the number of PHA molecules. This parameter facilitates a better understanding of our data.

In light of the above, the addition of PEG into culture medium causes a change in PHB-HV biosynthesis involving the enzyme system and results in the formation of a PHB-HV-PEG copolymer where the carboxylate (–COOH) terminus of PHB-HV chains are covalently linked by an ester bond to a PEG chain. PEG attachment to the PHB chain probably occurs during the synthesis of PHA polymers, suggesting possible interaction of PEG with PHB synthase enzyme and the polymer itself. The miscible nature of PEG with PHA and production of PHA/PEG blends was reported earlier [21], [45]. Previously it was shown that this reduction of PHB-HV molecular weight could be attributed to PEG limiting the polymer chain length [14]. PEG chain attachment with a covalent bond (resonance at 4.24 ppm) at the terminal position of a PHB chain could lead to break in the elongating PHA chain. The formation of low molecular weight PHB-HV-PEG copolymer by *Azotobacter chroococcum* may be attributed to the interaction of PEG with the PHA molecules itself, as was the case for *Ralstonia eutropha* [15]. This mechanism of PHB-HV-PEG copolymer synthesis can be confirmed by lower 3HV monomers content in the produced PHB-HV-PEG copolymer in comparison with PHB-HV copolymer, as shown in Table 1. When added in the medium 6 h after VA addition, PEG can cause inhibition of 3HV incorporation in the elongating copolymer chain.

### Physico-thermal Properties of Copolymers

**Figure 2:**
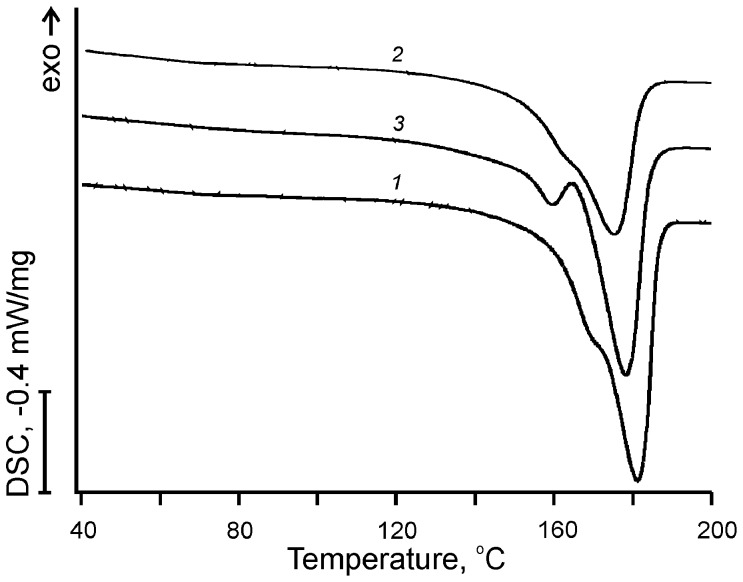
DSC thermograms of produced PHAs. DSC thermograms of produced biopolymers: (1) PHB homopolymer; (2) PHB–HV copolymer; and (3) PHB-PEG copolymer.

Introduction of 3HV into the PHA polymer chain caused significant changes in the physico-chemical characteristics of produced copolymers. We observed a decrease in crystallinity degree and melting temperature, as shown in Figure 2 and Table 1, which are in agreement with data in the literature [12]. DSC curves of Figure 2 revealed the thermal behavior of the polymers: PHB, PHB-HV and PHB-HV-PEG. All polymers were characterized by a melting peak typical of semi-crystalline polymers, as shown in Table 1. Analysis of DSC curves showed that HV incorporation in PHB chain caused: a) a great decrease in area of the PHA melting peak indicating a decrease in total crystallinity degree; b) a shift of the PHA melting peak to an area of lower temperature indicating a decrease in melting temperature, as shown in Figure 2 and Table 1. The PHB-HV-PEG copolymer had a lower total crystallinity degree and a lower melting temperature in comparison with PHB-HV, which coupled with decreased 3HV content in the PHB-HV-PEG copolymer, as shown in Figure 2 and Table 1. Unfortunately, the lower 3HV content in the PHB-HV-PEG masked a possible effect of PEG incorporation in PHB-HV polymer chain on the thermal properties of the copolymer. However, it should be noted, that the melting peak of PHB-HV-PEG endoterm is slightly split with the additional smaller peak at lower temperature (160°C). Possibly, the split peak, which indicated a less ordered packing of polymer chains in copolymer in comparison with homopolymer, can be connected with chemical structure of PHB-HV-PEG copolymer.

### Water Related Properties of Copolymers

The contact angles obtained on the PHB, PHB-HV and PHB-HV-PEG films of the polymers with different liquids (DEG, PEG 400, DMSO) were summarized in Table 2. The surface free energy components and total surface free energy were calculated from contact angles of water and other liquids and were shown in Table 3. Water absorbtion of polymer films also shown in Table 3 As seen in Table 1, despite the considerable change in physico-thermal characteristics of the PHB-HV copolymer relative to the PHB homopolymer, water-related properties (e.g., contact angle and water absorption) didn’t change. In contrast to the PHB-HV copolymer, the attachment of PEG to PHB-HV resulted in significant changes of water related properties, as shown in Table 1. The presence of PEG fragment in the PEG-containing copolymer resulted in a higher percentage of oxygen and hydrogen in the polymer, including decreased molecular weight along with increased water uptake capacity and hydrophilicity. Indeed, the water contact angle and water uptake parameters were significantly higher in PHB-HV-PEG: 12.8% decrease in contact angle and 7-fold increase in water absorption in comparison with PHB-HV. However, the calculated from contact angles total surface free energy of copolymers PHB-HV and PHB-HV-PEG only a little increased in comparison with PHB homopolymer and there is no difference in the total surface free energy between PHB-HV and PHB-HV-PEG copolymers.

**Table 2:** Contact angles of different liquids on polymer films.

	Water		DEG		PEG 400	
Polymer	“smooth” surface	“rough” surface	“smooth” surface	“rough” surface	“smooth” surface	“rough” surface
PHB	70.1±2.6	77.7±3.0	37.5±2.0	35.9±2.5	35.5±2.2	17.4±1.0
PHB-HV	70.4±2.3	77.8±3.6	37.7±2.2	42.5±2.6	37.3±2.6	16.2±1.6
PHB-HV-PEG	61.4±2.5* ^#^	77.2±3.3	38.1±2.1	41.7±2.2	32.3±2.4	48.6±2.3

* vs PHB, ^#^ vs PHB-HV, p<0.05.

**Table 3:** Surface free energy and water absorption of polymer films.

	Total surface free energy (γ_S_)	Surface free energy, dispertion component (γ_S_^d^)	Surface free energy, polar component (γ_S_^d^)	Water absorption (w/w, %)
Polymer	“smooth surface”			
PHB	41.1	29.8	11.3	0.6±0.2
PHB-HV	44.9	17.5	27.4	2.5±0.4*
PHB-HV-PEG	43.8	30.7	13.1	18.1±0.7* ^#^

* vs PHB, ^#^ vs PHB-HV, p<0.05.

### Surface Morphology of Copolymers Films

**Figure 3:**
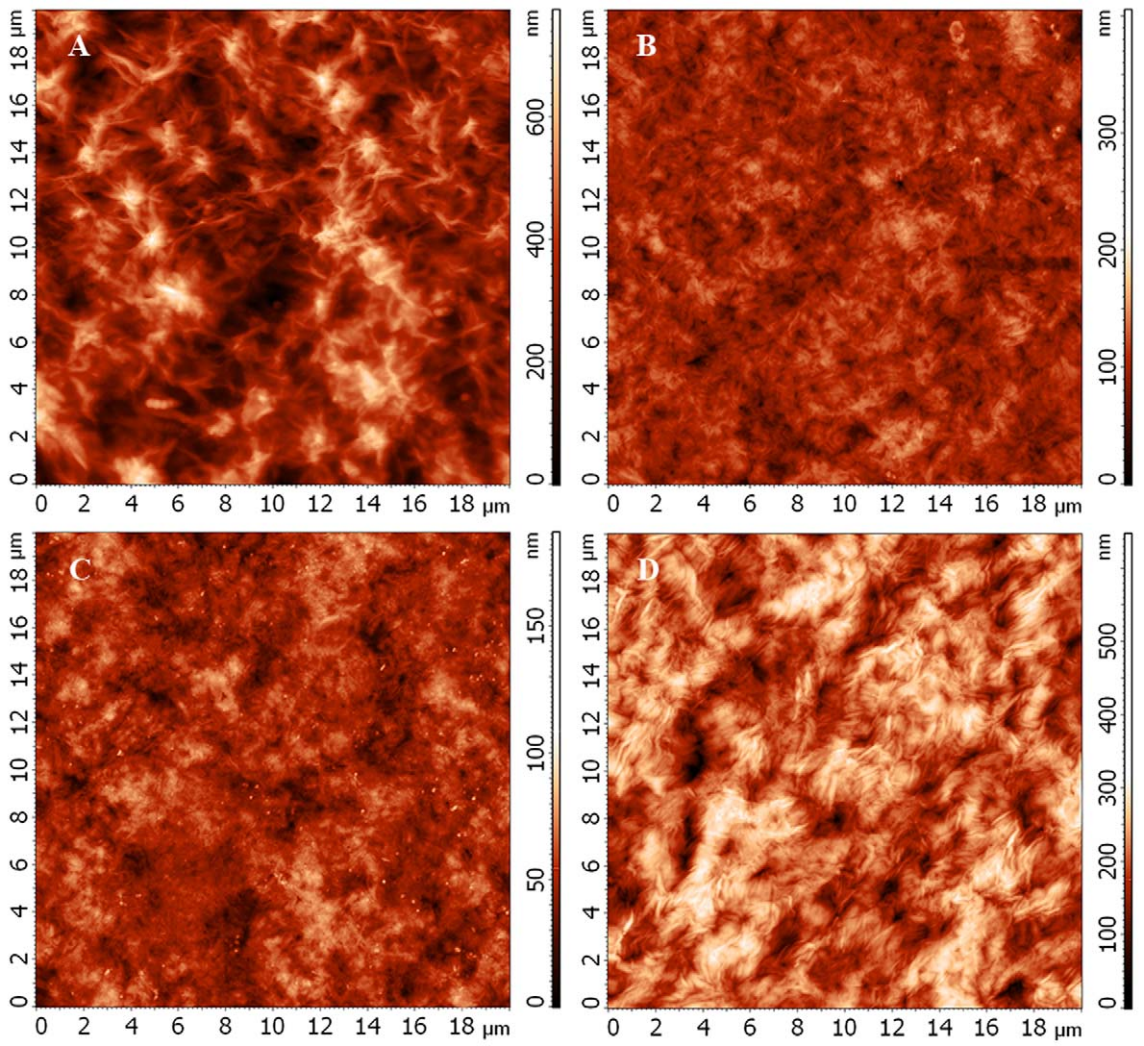
AFM microphotography of film surface of produced PHAs. AFM microphotography of film surface of produced PHAs: (a) PHB, “rough” surface; (b) “smooth” surface; (c) PHB-HV, “smooth” surface; (d) PHB-HV-PEG, “smooth” surface.

The film casting procedure allowed distinction of morphology between two surfaces when one plane of the polymer was adjacent to the glass plate and the other plane was exposed to air. Part a of Figure 3 clearly illustrates that the surface exposed to air has a roughness with plentiful pores characterized by a depth of 500–700 nm. As seen in Part b of Figure 3, the opposite side of the film that was in contact with the glass was characterized by minor texture and by shallower pores (as small as 100 nm). At higher magnifications (data not shown) in certain localities, the stacks of polymer crystallites with widths of about 100 nm and lengths between 500–800 nm were visible.

The variance of characteristics was related to solvent desorption conditions during its evaporation from the cast film. During chloroform evaporation from the air-exposed surface, the flux formed additional channels (viz. the pores), which were fixed as far as the film solidified and crystallized. Contrarily, during evaporation the morphology and texture of the opposite side of the film exposed to the glass support were not subjected to the impact of solvent transport. The morphology of the latter surface predominantly depended on energy interaction conditions (interface glass-biopolymer tension) [31].

The surface morphology of PHB and PHB-HV polymer films did not differ significantly, but the surface roughness of PHB-HV-PEG film was greatly increased. As seen in Table 4, the average and the root mean square roughness of both “smooth” and “rough” surfaces of PHB-HV-PEG terpolymer films increased significantly relative to the PHB homopolymer and PHB-HV copolymer.

**Table 4:** Roughness of polymer films surfaces.

Polymer	“smooth” surface		“rough” surface	
	R_a_, nm±SD	R_q_, nm±SD	R_a_, nm±SD	R_q_, nm±SD
PHB	5.5±1.3	7.3±1.7	92±7	115±12
PHB-HV	8.1±1.8	11.8±2.6	73±8*	93±11
PHB-HV-PEG	30.9±2.3* ^#^	39.1±2.9* ^#^	147±36* ^#^	190±47* ^#^

Average roughness (R_a_) and the root mean square roughness (R_q_) of PHB, PHB-HV, and PHB-HV-PEG films surface.

* vs PHB, ^#^ vs PHB-HV, p<0.05.

The average roughness of “smooth” surface of PHB-HV-PEG film was almost four-fold higher than PHB-HV film and more than five-fold higher relative to the roughness of PHB film. Microstructure formation on the polymer film surface exposed to the glass surface (e.g., packing of polymer chains) depended on the crystalline/amorphous ratio of polymer matrix and glass-biopolymer interaction, which was also connected to the water-related properties of the polymer [46]. But the average roughness of “rough” surface of PHB-HV-PEG film was also significantly increased in comparison with PHB and PHB-HV. Thus, the difference in film surface roughness can be determined by a combination of crystallinity and hydrophobicity of polymers. Indeed, crystallinity of PHB copolymers was lower than PHB, and the hydrophilicity of PEG-modified copolymer was higher than PHB and PHB-HV. These factors can lead to a less ordered laying of polymer structures and consequently, an increase in copolymer surface roughness.

### Protein Adsorption

**Figure 4:**
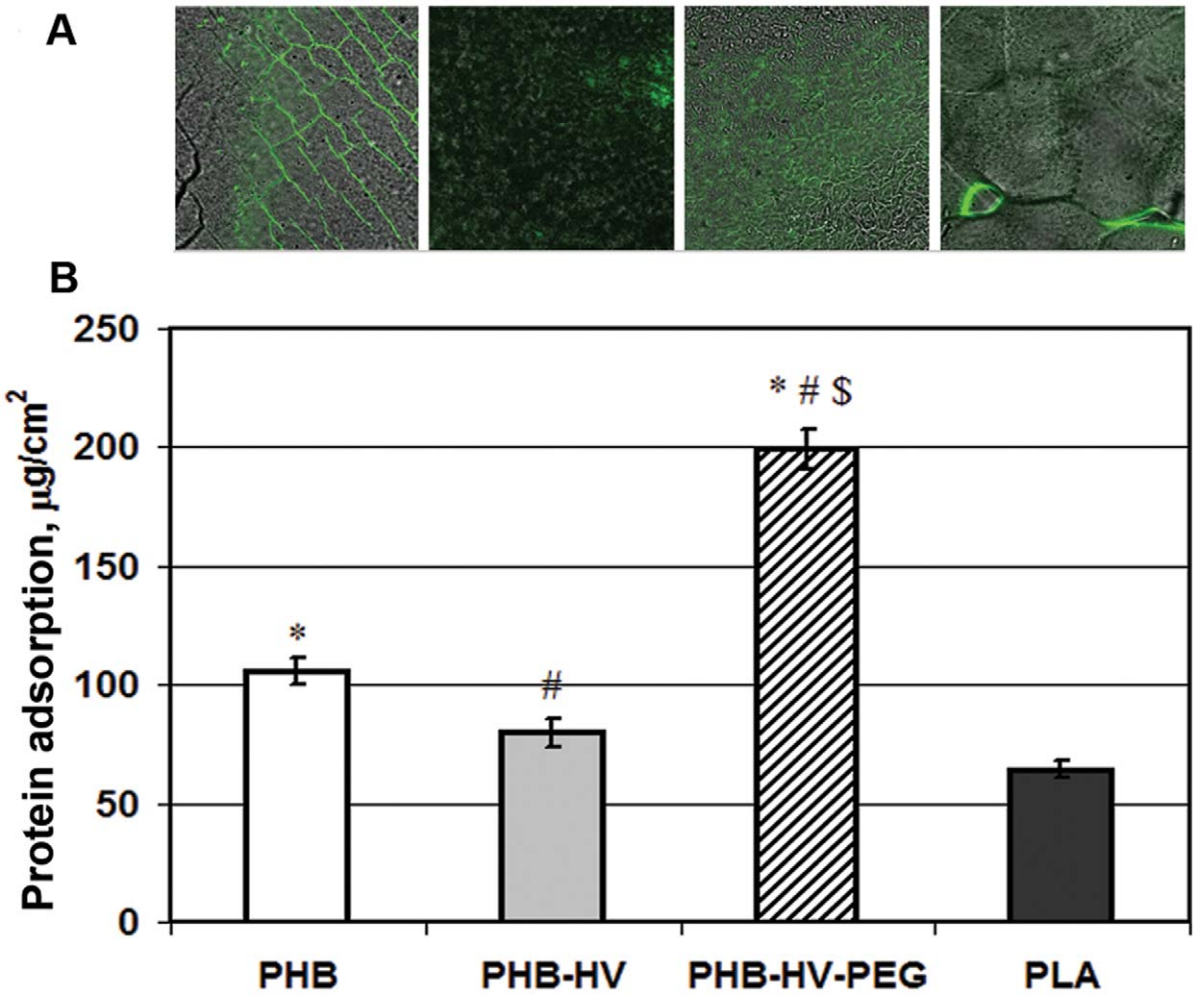
The protein adsorption on the polymer surface. The protein adsorption on the polymer surface: **(**a) FITC-BSA distribution on the surface of PHB, PHB-HV, PHB-HV-PEG, and PLA polymer films visualized by florescence microscopy; (b) the absorption of proteins from bovine fetal serum on the PHB, PHB-HV, PHB-HV-PEG and PLA polymer films. Data were shown as Mean±SD (n = 6); ^*^ vs PLA, ^#^ vs PHB, ^$^ vs PHB-HV, p<0.05.

Figure 4 shows the protein adsorption, which was detected on the films of PHB, PHB-HV and PHB-HV-PEG. Films from PHB-HV-PEG presented the highest protein absorption ability approximately 2-fold greater than PHB and PHB-HV films, as shown in Figure 4b. This result is in agreement with the observation made by Collier et al. [47] that albumin adsorbs preferably to hydrophilic surfaces, which seems to correlate with the wettability results of PHB-HV-PEG terpolymer films (Table 1). A positive correlation between polymer surface hydrophilicity and protein adsorption to polymer surface was shown also in other studies [33], [48]. Moreover, analysis of protein adsorption by fluorescence microscopy demonstrated the difference in morphological distribution of adsorbed FITC-BSA on the polymer surface. As seen in Figure 4a, on the PHB films surface the protein adsorbed greatly onto defects of polymer surface, while adsorption of FITC-BSA on PHB-HV and PHB-HV-PEG films surface was uniform. The uniform distribution of adsorbed protein on the PHB-HV and PHB-PEG film surface is associated probably with decreased crystallinity of the polymers, while the greater amount of adsorbed protein on the PHB-HV-PEG film compared to PHB-HV film is associated with greater PHB-PEG surface hydrophilicity.

### Biocompatibility of Produced Biopolymers In Vitro

**Figure 5:**
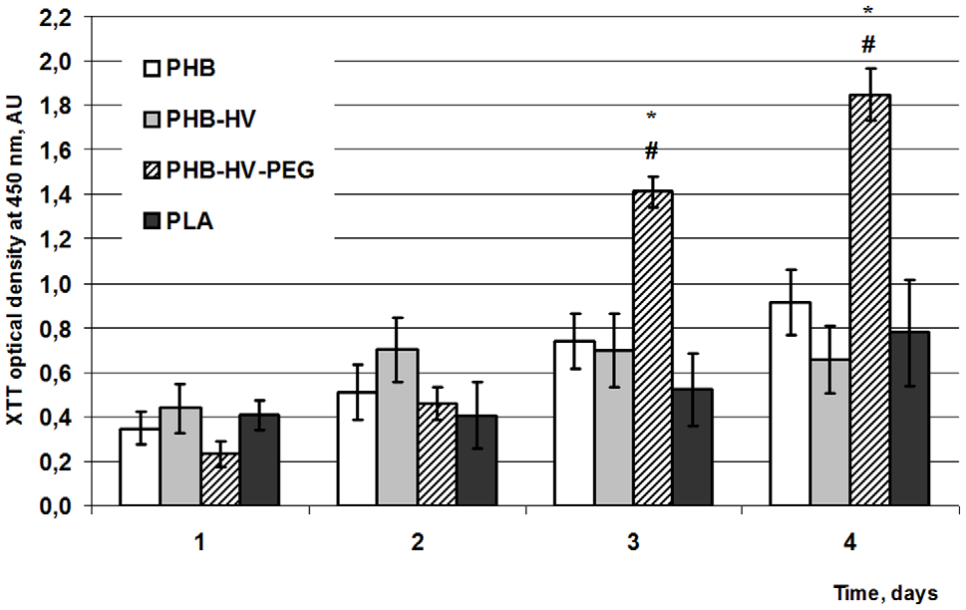
Adhesion and cell proliferation on tested biopolymer films. Adhesion and cell proliferation on tested biopolymer films evaluated by XTT test: PHB, PHB-HV, PHB-HV-PEG, and PLA (^*^ vs PLA, ^#^ vs PHB, ^$^ vs PHB-HV, p<0.05).

Cell cytotoxicity testing is one of the critical factors affecting the biomedical application of polymers [1], [2]. Here, we used 3T3 fibroblasts to demonstrate that the naturally hydrophobic PHB-surface could be modified into a more cell-compatible surface by 3HV and PEG modification of the PHB biopolymer. Cells exhibited remarkable growth and proliferation after only 24 h incubation on different polymers films compared with PLA (control), as measured by the XTT assay. Cell adhesion of cells on PHB films showed a tendency to be stronger than on PLA films at 2–4 days, but this difference was not significant. There was also no significant difference in cell adhesion between the PHB-HV and PLA as well as between the PHB-HV and PHB. However, as indicated in Figure 5, there was a distinct difference between the cell attachment on the PHB homopolymer and PEG-modified PHB-HV copolymer. Figure 5 illustrates that 3T3 fibroblasts attached to tested PHB-HV-PEG films displayed significantly stronger adhesion compared to their interaction with PHB, PHB-HV and PLA films at 3 and 4 days incubation. After 4 days incubation, the highest XTT values were observed with PHB-HV-PEG film, which was almost equal to that of polystyrene plate, whereas cells grown on other tested films showed cell adhesion approximately two to three-fold lower than that of TCPs (data not shown).

As commonly known, protein adsorption plays an important role in cell adhesion. Generally, cells grow on a layer of protein that interacts with cellular receptors and the hydrophilic surface is favorable to adhesion and growth of cells [33], [49]. The more hydrophilic surface of PHB-HV-PEG films facilitated absorption of proteins. Indeed, PHB-HV-PEG displays more hydrophilic properties (that were evaluated by measurement of water contact angle and water uptake parameters) relative to PHB and PHB-HV. Moreover, as seen in fluorescent microscopy microphotographs of Figure 4a, irregular protein adsorption on PHB films can hinder protein layer formation as opposed to PHB-HV-PEG film, which is covered with protein uniformly. However less surface hydrophilicity and irregular protein adsorption of PHB are related to “smooth” surface of polymer films and can influence on cell adhesion to “rough” surface only indirectly. But the highest water absorption, as indicated in Table 3, and total protein adsorption on terpolymer films, as indicated in Figure 4b, can have a great influence on cell growth on “rough” surface of films. Indeed, cell adhesion of 3T3 fibroblasts to more hydrophilic (compared with PHB) PEG-modified PHB copolymer was stronger in opposition to cell adhesion to more hydrophobic (compared with PHB) PHB-HV copolymer. This data correlates with biocompatibility of PHB/PHB-HHx blends, which depends on surface hydrophilicity of polymer films [33]. However, not only surface hydrophilicity but also surface morphology effects cell attachment and proliferation. Different cells prefer different surfaces, e.g., it was found that fibroblasts preferred to attach to a relatively rougher surface, while epithelial cells only attached to the smoothest surface [50]. The 3T3 cells grew on the “rough” surface of polymer films, the roughness of this surface of PHB-HV-PEG terpolymer was significantly 2-fold higher in comparison with PHB-HV, as shown in Table 4. Possibly a rougher surface of PHB-HV-PEG film is also responsible for the significantly stronger cell adhesion to PHB-HV-PEG film in comparison with the PHB and PHB-HV films. Moreover, the stimulating effects of surface hydrophilicity and roughness of the terpolymer on the cell attachment and proliferation could be summarized. Thus, our results demonstrate that the PHB-HV-PEG terpolymer possesses the ability to maintain cell viability and growth, thereby indicating the non-toxicity of the PEG-modified PHB-HV copolymer to 3T3 fibroblasts.

### Conclusions

Taken together, our results indicate that introduction of VA and PEG into A. chroococcum 7B culture represent a viable approach to the production of PHB-HV-PEG terpolymer with significantly changed physico-chemical properties. Despite of low EG-monomers content in bacterial-origin PHB-HV-PEG polymer, the terpolymer demonstrated great improvement in biocompatibility in vitro in contrast to PHB and PHB-HV copolymers, which may be coupled with increased protein adsorption, hydrophilicity and surface roughness of PHB-HV-PEG terpolymer. Currently, we are working to adjust the materials by tailoring the compositions achieve a balance between biocompatibility, physicochemical properties, processing ability and device fabrication.
